# A Theoretical Analysis for Assessing the Variability of Secondary Model Thermal Inactivation Kinetic Parameters

**DOI:** 10.3390/foods6010007

**Published:** 2017-01-12

**Authors:** Maria C. Giannakourou, Nikolaos G. Stoforos

**Affiliations:** 1 Department of Food Technology, Technological Educational Institute of Athens, Athens 12210, Greece; mgian@teiath.gr; 2 Department of Food Science and Human Nutrition, Agricultural University of Athens, Athens 11855, Greece

**Keywords:** *D_T_* and *z*-values, Monte Carlo, kinetics, confidence intervals, error propagation

## Abstract

Traditionally, for the determination of the kinetic parameters of thermal inactivation of a heat labile substance, an appropriate index is selected and its change is measured over time at a series of constant temperatures. The rate of this change is described through an appropriate primary model and a secondary model is applied to assess the impact of temperature. By this approach, the confidence intervals of the estimates of the rate constants are not taken into account. Consequently, the calculated variability of the secondary model parameters can be significantly lower than the actual variability. The aim of this study was to demonstrate the influence of the variability of the primary model parameters in establishing the confidence intervals of the secondary model parameters. Using a Monte Carlo technique and assuming normally distributed *D_T_* values (parameter associated with a primary inactivation model), the error propagating on the *D_Tref_* and *z*-values (secondary model parameters) was assessed. When *D_T_* confidence intervals were broad, the secondary model’s parameter variability was appreciably high and could not be adequately estimated through the traditional deterministic approach that does not take into account the variation on the *D_T_* values. In such cases, the proposed methodology was essential for realistic estimations.

## 1. Introduction

Processing and storage induce changes in foods due to biological, chemical and physical reactions. Such changes proceed at a certain rate and with particular kinetics. Kinetic modeling enables us to describe these changes and their rates quantitatively and has become a very useful approach in relation to food processing and food quality. Significant published work regarding kinetic modeling has focused on microbiological safety and spoilage in a variety of food matrices [1] or on describing basic reaction mechanisms that can be important for quality evaluation and control [2].

Food kinetics is based on the thorough study of the rates at which physico- and bio-chemical reactions proceed. The area of kinetics in food systems has received a great deal of attention in past years, primarily due to efforts to optimize or at least maximize the quality of food products during processing and storage [3]. The use of chemical kinetics, the study of the rates and mechanisms that are involved in the reactions of interest, and the mathematical relationships that best describe the influence of different external or internal factors, such as pH, water activity, pressure, or in particular temperature, on the reaction rate constants have been used to model changes in food quality. Kinetic parameters describing such changes are needed.

When building mathematical equations to describe food kinetics, experimental data is used to estimate relevant parameters. Both for microbiological and chemical predictions, the choice of conditions, the experimental design, as well as the quality of experimental, data is important for the validity of parameters, and hence the reliability of the prediction [4]. The estimation of model parameters per se, and the accurate calculation of their variation (expressed by its confidence intervals) affect considerably the final predictions. To obtain the best results, careful choice of primary and secondary models as well as appropriate statistics are necessary.

The most widely applied procedure for food kinetic modeling (either for microbiological measurements or for chemical changes) includes a step-to-step procedure [5,6,7,8,9,10,11,12]. In such an approach, initially, experiments are conducted under different constant temperature conditions, the most representative indices are selected, and their change is measured as a function of processing or storage time. From this point, the traditional two-stage methodology is based on the application of an appropriate primary model in order to describe the rate of these changes and determine, through appropriate mathematical equations, the relevant kinetic parameters for each temperature. The second step is to select a secondary model that best describes the effect of temperature on the respective kinetic parameters. The ultimate goal is to build this secondary model and define not only the estimates of its parameters, but also provide information on their variability. Consequently, knowledge of all parameters of primary and secondary models would allow for the prediction of quality or microbiological status of the food at any, different temperature of interest. If validated, mathematical models can be a useful tool to assess safety and quality after processing and at any stage of food storage. This conventional method of a two-step procedure for food modeling is however very time-consuming and lacks reliability concerning the expression of parameters’ variability [4]. In its present form, errors, or equivalently, confidence intervals of primary model parameters at each temperature are not taken into account when calculating secondary model parameters. As a consequence, confidence intervals of estimates of the kinetic parameters of the secondary model can be falsely narrower than the actual confidence intervals, implied by the experimental data. There are numerous published studies that ignore the variation in the primary model’s kinetic parameters while reporting confidence intervals for the parameters appearing in the secondary model [6,12,13,14,15,16,17,18,19,20,21,22,23,24,25,26,27,28]. In fact, this has become the standard procedure in the literature. In such cases, either a first or an *n*th reaction order equation or some other inactivation model, or a mathematical expression to describe microbiological growth (for example Gompertz, Baranyi, logistic etc.) is selected according to the results of the best fit criterion applied in each experimental data set. Thereafter, Arrhenius equation, amongst numerous models proposed in current literature, is mostly used as a secondary model to describe the temperature effect on kinetic parameters. In all these studies, regardless of the primary and secondary model used, and no matter the index measured or the food matrix in question, secondary model kinetic parameters’ uncertainty (expressed as confidence intervals of the parameter) does not incorporate primary model parameters’ true variation.

A reliable alternative is deemed necessary to account for the real uncertainty of model parameters, in order to be able to predict, in a more accurate way, chemical or microbiological changes, at any selected temperature and time during processing and product shelf life (at storage or distribution). The Monte Carlo simulation technique has been recently proposed in literature for the probabilistic assessment of stochastic variability and uncertainty associated with microbiological [9,29,30,31,32] or chemical/sensory [33,34,35,36,37,38] attributes of several food matrices.

The Monte Carlo technique is also used as a background tool in the bootstrap methodology described by [39] in 1993, first applied in scientific fields other than food science. Recently, a bootstrap Monte Carlo analysis has been proposed to compute the variation of the primary model’s parameters based on the propagation of error, artificially introduced to the experimental data (concentration vs. time data); it was assumed that the experimental data was normally distributed and the error introduced at each measurement was based on its standard deviation [40,41,42,43,44]. However, the variation of the primary model parameters has not been taken into account in determining the uncertainty of the parameters of the secondary model. Therefore, the scope of this work was to assess the propagation of the primary model parameters variability into the secondary model kinetic parameters estimation. As a case study, thermal inactivation data of l-carnitine under isothermal conditions was used along with the classical thermobiological approach [45,46] for the determination of the respective parameters of the primary (decimal reduction time, *D_T_*) and secondary (*z* and *D_Tref_* values) models. For this purpose, Monte Carlo simulation, designed and implemented through a FORTRAN algorithm was used.

## 2. Materials and Methods

According to the classical thermobacteriological approach for thermal inactivation kinetics [45,46], the inactivation rate is described by the decimal reduction time (*D_T_*), through the following Equation (1):

(1) $$\log C=\log C_{o}-\frac{t}{D_{T}}$$

where *D_T_* is defined as the time in minutes, at constant temperature, required to reduce the initial concentration of a thermolabile substance by 90%, *C* the concentration at time *t* and *C_ο_* the initial concentration of the thermolabile substance.

Based on Equation (1) and the application of linear regression, the *D_T_* value is determined at each temperature of the isothermal experiments, from the negative reciprocal of the corresponding slope. The temperature dependence of *D_T_* is characterized by the *z* value, that is, the increase in temperature necessary to induce a 10-fold reduction in *D_T_* (Equation (2)):

(2) $$\log D_{T}=\log D_{T_{ref}}+\frac{T_{ref}-T}{z}$$

where *Τ_ref_* is a reference temperature. Parameter *z* is determined as the negative reciprocal of the slope of log *D_T_* vs. *T* regression line.

### Monte Carlo Simulation

The proposed procedure was based on the assumption that the estimate of the main parameter *D_T_* of Equation (1), which is determined by linear regression of log*C* vs. *t* data for each temperature, can be described by a distribution of values rather than by a single value. The benefit of this approach is that it incorporates the error in *D_T_* calculated by the linear regression of the primary model, and thus the variability of the parameter [47,48]. For the development of this methodology, experimental data of l-carnitine thermal degradation, describing its inactivation at eight different constant temperatures were used [49].

**Figure 1:**
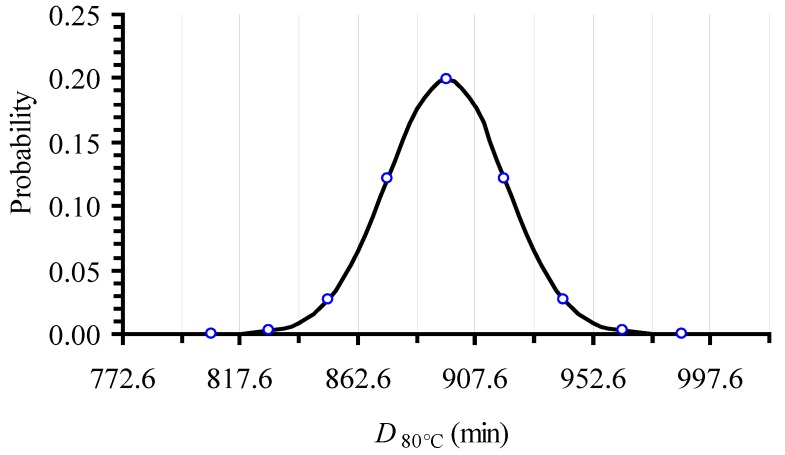
Normal distribution of *D*_80 °C_ value, based on the mean value (896.3) and the standard deviation (22.5) of the isothermal log*C* vs. *t* inactivation data of l-carnitine at 80 °C and the corresponding linear regression analysis. Vertical lines are designed to be 0.5 × *σ* apart.

In applying the Monte Carlo simulation, a normal distribution was assumed to describe *D_T_* variability. A similar approach was used by [37] when assessing the confidence intervals of *E_a_* and *k_ref_* parameters of the Arrhenius equation for non-isothermal degradation of cyanidins. An example of such a normal distribution, which is constructed based on the estimate of the mean value, *μ*, of 896.3 min and its standard deviation, (*σ* = 22.5 min) is demonstrated in Figure 1; it refers to the *D*_80 °C_ value and it is based on the relative experimental data. The distribution was discretized every 0.5 × *σ* intervals on the *D_T_* scale, the central interval being located between *μ −* 0.25 × *σ* and *μ* + 0.25 × *σ*. Within each such interval, a single *D_T_* value, equal to the *D_T_* value at the middle of the particular interval, was assigned (Figure 1). The same procedure was followed to construct the corresponding distribution curves for all eight temperatures of the isothermal experiments.

**Figure 2:**
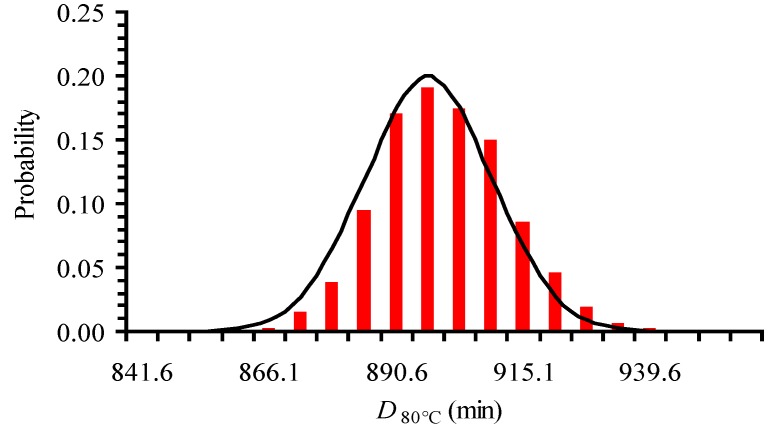
Histogram of *D*_80 °C_ data generated based on 1000 log*C* vs. time synthetic data sets showing approximately normally distributed values.

There are some (limited) publications that refer to the distributions that best describe primary models’ kinetic parameters. For example, Poschet et al. [42], in a Monte Carlo analysis, represented the parameter distributions of a microbial growth model by normal distributions. Results by Huang and Vinyard [9] showed that the distributions of a primary model (a system of ordinary differential equations) parameters were approximately normal. Pénicaud et al. [50] superimposed a pseudo random noise on the experimental data, performed 2000 runs using Monte Carlo technique and showed that the primary model’s parameters (apparent reaction rate constant of ascorbic acid loss) were normally distributed. Our assumption about *D_T_* having a normal distribution was checked by creating 1000 log*C* vs. time synthetic data sets at 80 °C, estimating *D*_80 °C_ for each set, and finally plotting a histogram of *D_T_* values (Figure 2). The synthetic data sets were based on the experimental measured *C* data and their variation, by assuming that they were normally distributed and using a Monte Carlo technique implemented in a FORTRAN coded program. Based on this plot, where the normal distribution line was also added (Figure 2) we concluded that *D_T_* values were approximately normally distributed. As a consequence, *D_T_* values can be described by a mean value and a symmetric standard deviation.

The Monte Carlo technique [37,51] was implemented through a FORTRAN based algorithm. At each iteration, a random number was generated out of a normal distribution and a value was assigned to *D_T_* based on the discretization of the corresponding normal distribution curve. The correlation between the random numbers and the respective values of *D_T_* was based on the frequency determined by the corresponding distribution, for each of the eight temperatures studied. In our study, Monte Carlo simulation was repeated 500 times, assigning 500 values to each *D_T_* parameter, for all eight temperatures studied. For example, in the case of the distribution curve for the 80 °C case, out of the 500 randomly selected *D_T_* values, 100 were assigned with the mean value of 896.3 min, giving a percentage of 20%; 60 were given a value of 873.9 min, giving a percentage of 12%; five were given a value of 840.1 min, giving a percentage of approximately 1% and so on, as shown in Figure 1. Thus, 500 sets of eight *D_T_* values, each one at each of the eight experimental temperatures, were created.

The ultimate goal was to determine the values and the corresponding errors (or confidence intervals) of *D_Tref_* and *z* (Equation (2)) based on these 500 *D_T_* datasets that have been produced out of the previous step of Monte Carlo application. For each specific, random, set of the eight *D_T_* values, a pair of estimates for *D_Tref_* and *z* and their corresponding variations was assessed based on linear regression of log *D_T_* vs. T data. For the 500 pairs of *D_Tref_* and *z* values and their assumed normal distribution (created based on the estimated regression 95% confidence intervals) and using again the Monte Carlo technique, 500 *D_Tref_* and *z* values were randomly chosen from each distribution. The final set comprised 500 × 500 values for each parameter of the secondary model, *D_Tref_* and *z*. From these 500 × 500 values for each parameter, the mean values and the corresponding 95% confidence intervals of *D_Tref_* and *z* parameters were calculated. At this point, it should be noted that, as a reference temperature, the value of 120 °C was used.

Using the error propagation laws, generally described by Equation (3) [52,53] and in order to calculate the 95% Confidence Intervals (CI) of the parameters *D_T_*, *D_Tref_* and *z* (described as δ(*D_T_*), δ(*D_Tref_*), δ(*z*), respectively) from the corresponding 95% CI of 1/*D_T_*, 1/*z*, and log(*D_Tref_*) (described as δ(1/*D_T_*), δ(log(*D_Tref_*)), δ(1/*z*), respectively) appearing in Equations (1) and (2), Equations (4)–(6) were used:

(3) $$\delta \left(f(x)\right)=\left|\frac{\partial f(x)}{\partial x}\;\delta \left(x\right)\right|$$

where *δ*(*f*(*x*)) is a measure of the error/uncertainty (for example, expressed as the 95% CI) of an *f*(*x*) function.

(4) $$\delta \left(D_{T}\right)=\frac{1}{{\left[\frac{1}{D_{T}}\right]}^{2}}\;\delta \left(\frac{1}{D_{T}}\right)$$

(5) $$\delta \left(z\right)=\frac{1}{{\left[\frac{1}{z}\right]}^{2}}\;\delta \left(\frac{1}{z}\right)$$

(6) $$\delta \left(D_{T_{ref}}\right)=\ln(10)\;D_{T_{ref}}\;\delta \left(\log(D_{T_{ref}})\right)$$

## 3. Results and Discussion

**Figure 3:**
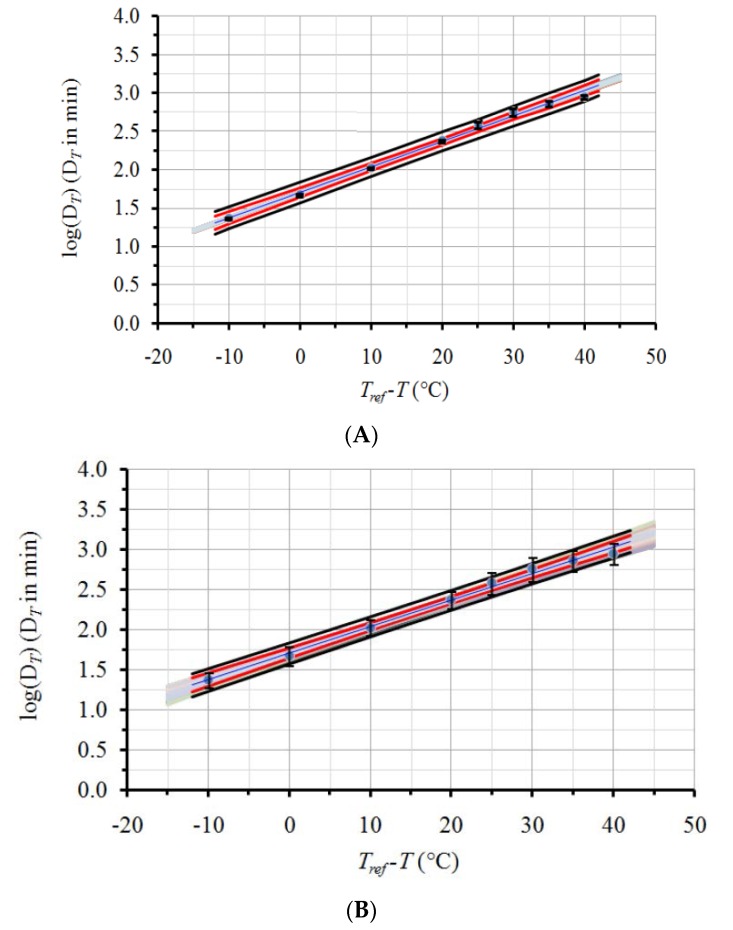
Effect of *D_T_* data uncertainty on secondary model predictions. Circles depict mean values and error bars represent the ±95% CI for *D_T_* data with (**Α**) original variation; and (**Β**) expanded error. Blue straight line is the linear regression line of the mean values; red lines represent the ±95% Confidence Band (CB) and black lines represent the ±95% Prediction Band (PB). Grey line zone shows regression lines for 250 indicative *D_T_* sets, produced by Monte Carlo simulation.

The *D_T_* values, obtained through Equation (1) and the experimental data of l-carnitine thermal degradation, and the corresponding 95% Confidence Intervals (CI) are depicted on the second and third columns of Table 1, respectively. Based on these *D_T_* values and linear regression through Equation (2), the following estimates, and the corresponding ±95% CI, were calculated for the secondary model parameters: *z* (in °C) = 30.2 ± 2.3 and *D_Tref_* (in min) = 50.6 ± 7.3 for a chosen *Τ_ref_* equal to 120 °C. The application of the proposed methodology resulted on the same mean *D_Tref_* and *z* values and did not lead to significant changes of the 95% CI of the parameters *D_Tref_* and *z* compared to their initial estimates. The 95% CI on the *D_Tref_* and *z* found through the proposed analysis were ±7.7 min and ±2.4 °C, respectively, comparable to the initial estimates of ±7.3 min and ±2.3 °C, respectively, that were calculated without taking into account the uncertainty at the determination of each *D_T_* value (at each temperature). This was attributed to the relatively low error (narrow 95% CI) of *D_T_* values at each of the temperatures of the experiment compared to the error of the linear regression (Figure 3a). Consequently, the described methodology was tested using a higher, arbitrarily decided error on the same, experimentally calculated mean *D_T_* values (Table 1, fourth column). Thereafter, the procedure previously described was repeated with the new, expanded ±95% CI of *D_T_* values. The legitimacy of the procedure, as far as the Monte Carlo scheme used and the randomness of the selections, was confirmed by the estimation of the mean values and the 95% CI of the 500 random sets of the *D_T_* values and the resulting proximity to their initial values (Table 1).

In order to better visualize the importance of the initial variability of *D_T_* values on the secondary model kinetic parameters, a qualitative illustration is given in Figure 3. In this figure, original data points (log(*D_T_*) vs. *T_ref_-T*), the linear regression line and respective 95% confidence bands (CB, red lines) and prediction bands (PB, black solid lines), are indicated. Error bars represent the ±95% CI for the *D_T_* data. Figure 3a refers to the data with the original, narrow 95% CI (columns 2 and 3 of Table 1) while Figure 3b uses the data with the intentionally expanded 95% CI (columns 2 and 4 of Table 1). As it can be seen, regression, ±95% CB and ±95% PB lines are identical for both Figure 3a,b. The regression line uses only mean *D_T_* values. The equations also used for the construction of the ±95% CB and ±95% PB (Equations (7) and (8), respectively) do not include any *D_T_* variation in their formulations. For *y* = log(*D_T_*) and *x* = (*T_ref_*-*T*), the expected value of y for a given *x_o_* (±95% CB) is given by [52]:

(7) $${\overset{⌢}{y}}_{o}\pm t_{a/2}\;\sigma_{y}\;\sqrt{\frac{1}{N}+\frac{{\left(x_{o}-\bar{x}\right)}^{2}}{\sigma_{xx}}}$$

while the predicted value of y for a given *x_o_* (±95% PB) is given by:

(8) $${\overset{⌢}{y}}_{o}\pm t_{a/2}\;\sigma_{y}\;\sqrt{1+\frac{1}{N}+\frac{{\left(x_{o}-\bar{x}\right)}^{2}}{\sigma_{xx}}}$$

where *σ_y_* is the standard deviation of the residuals, *σ_xx_* is the sum of squared error, N is the number of variables (here equals eight, the number of experimental temperatures), ${\overset{⌢}{y}}_{o}$ is the estimated value of *y* based on linear regression, $\bar{x}$ the mean value of all *x*’s and *t*_a/2_ is the value of Student distribution for N-2 degrees of freedom.

In Figure 3a,b, the straight grey lines included represent the linear regression lines for 250 (out of the 500) sets of *D_T_* values, both for the original narrow 95% CI (Figure 3a), as well as for the intentionally expanded 95% CI (Figure 3b) of our case study. It can be observed that the range and error bars of these log(*D_T_*) vs. (*T_ref_* − *T*) regression lines, are much wider in Figure 3b, confirming the expanded variability. Whereas in Figure 3a, grey lines lie well within the ±95% CB (due to their small experimental error), in Figure 3b, grey lines’ range not only exceed the ±95% CB but almost cover the whole ±95% PB, owing to the intentionally expanded error of the experimental data.

To demonstrate the rationale of the proposed approach, let us consider an extreme scenario, where the experimentally determined mean values of *D_T_* parameter for the eight temperatures were lined in a perfect straight line, giving a linear regression with *R*^2^ = 1 and σ_xx_ = 0. In that case, all regression, ±95% CB and ±95% PB lines would coincide regardless of the variability of the primary model’s parameter (*D_T_*). This would easily lead to false interpretation of secondary model parameter estimates and poor predictions. In this extreme case, as well as in the case of the expanded error studied in this work, application of the proposed methodology can be of significant help to estimate realistically the variability of secondary model’s parameters.

**Figure 4:**
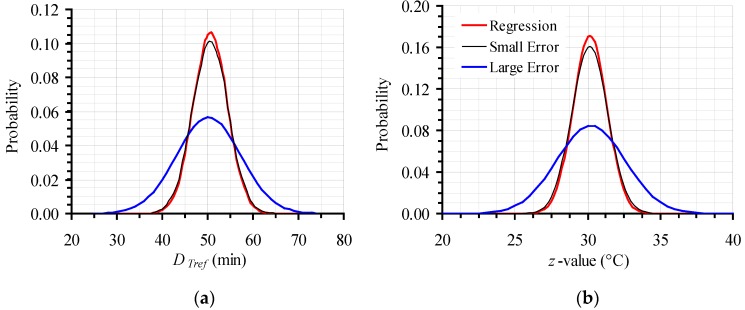
Distribution of the 500 × 500 values generated by the Monte Carlo simulation, based on the proposed methodology for the initial, narrow (Small Error) and the expanded (Large Error) 95% CI, compared to the results of the regression analysis of the original data for (**a**) *D_Tref_*; and (**b**) *z* value.

Mean values and standard deviations of the *D*_120 °C_ and *z* values (500 × 500 data points for each parameter) generated by Monte Carlo simulations were calculated and used to create the corresponding frequency curves, both for the original, narrow, as well as for the intentionally expanded 95% CI, for both kinetic parameters, *D*_120 °C_ and *z* (Figure 4a,b). Estimates (mean values) and their variation (represented by standard deviation) of secondary model kinetic parameters (*D_Tref_* and *z*-value), as calculated by regression analysis for the initial experimental data, were used to build the corresponding frequency curve (Figure 4). The curve, based on regression analysis estimates (both its height, as well as its broadness), was in proximity to the results of the Monte Carlo simulation, when the narrow 95% CI were used (as expected from Figure 3a depiction). In the case of the expanded error, however, the respective frequency curve, based on values generated by the Monte Carlo simulation, differed significantly from the distribution based on the calculations of regression analysis, showing the necessity of the introduced approach.

The distributions in Figure 4a,b were constructed based on mean values and their 95% CI of parameters *D*_120 °C_ and *z* respectively, which were calculated to be 50.2 ± 13.9 min and 30.2 ± 4.6 °C, for the expanded error, compared to 50.6 ± 7.7 min and 30.2 ± 2.4 °C for the small, original error.

**Figure 5:**
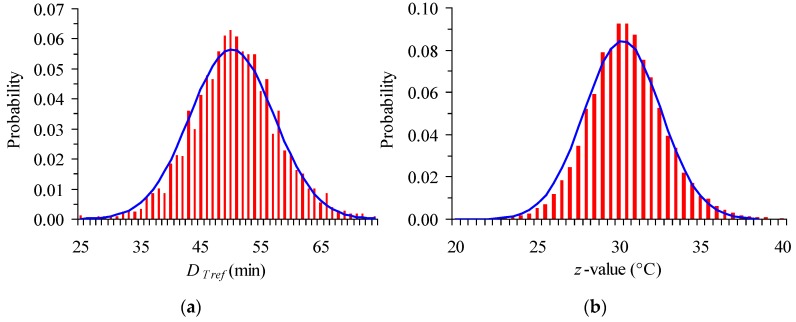
Histograms of (**a**) *D_Tref_* and (**b**) *z* values generated by Monte Carlo simulation, for the expanded 95% Confidence Interval (CI) (together with the corresponding normal distributions).

It is a common practice that estimation of model parameters is expressed as mean ± standard deviation, or mean ±95% CI, which implicitly assumes normal distributions of the corresponding parameter. In our case study, the *D_T__ref_* and *z* values out of the Monte Carlo iterations were presented in histograms (a number up to 50,000 iterations was used) and shown to be adequately described by a normal distribution (Figure 5). Thus, CI can be considered as symmetric.

**Figure 6:**
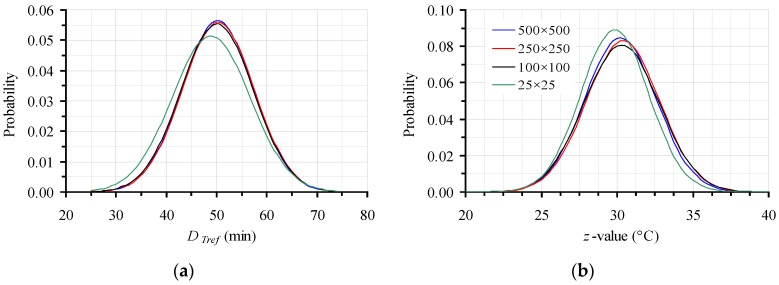
Effect of the number of points generated by Monte Carlo simulation, on the distribution of (**a**) *D_Tref_* and (**b**) *z* values for the data with the intentionally expanded 95% CI.

All the discussion up to this point was made on a 500 × 500 data points’ base for Monte Carlo simulation. However, before selecting the appropriate number of trials, a sensitivity analysis was performed for both parameters of the secondary model (*D*_120 °C_ and *z*), which, for the sake of clarity was not presented earlier. This study, again realized using a FORTRAN code, is deemed necessary in order to test Monte Carlo effectiveness and seek the necessary repetitions to obtain a reliable approximation of the “real” confidence intervals and thus, parameters’ variability. In this context, four distinctive cases of a significantly different number of trials were implemented, namely 25 × 25, 100 × 100, 250 × 250 and 500 × 500, following the procedure previously detailed. The estimated mean values and corresponding 95% CI for the *D*_120 °C_ (in min) and *z* (in °C) values were 48.9 ± 15.2 and 29.8 ± 4.4, 50.1 ± 14.1 and 30.3 ± 4.8, 50.3 ± 14.0 and 30.4 ± 4.7, and 50.2 ± 13.8 and 30.2 ± 4.6, for the cases of 25 × 25, 100 × 100, 250 × 250 and 500 × 500 data sets, respectively. These results (Figure 6) indicated that, excluding the 25 × 25 iterations simulation, the use of only 100 points out of each of only 100 distributions of *D*_120 °C_ and *z* (based on random 100 data sets of *D_T_* values of the primary model) is sufficient to approximate the realistic variations of the kinetic parameters of the secondary model. However, since the computing power and speed nowadays is easily available without big costs, the application of at least 250 × 250 trials is preferable to attain more accurate predictions.

## 4. Conclusions

An approach that takes into account the uncertainty of the primary model rate parameters of thermal inactivation (*D_T_* values) and integrates it in the variability of the secondary model parameters (*D_Tref_* and *z*) was implemented in a FORTRAN code through a Monte Carlo scheme. When *D_T_* confidence intervals were broad, the secondary model’s parameter variability was appreciably high and could not be adequately estimated through a traditional deterministic approach. In such cases, the proposed methodology was essential for realistic estimation of the uncertainty on secondary model parameters. For the example presented, the 95% CI on the mean *D_Tref_* and *z* values estimated with the proposed procedure were ±13.9 min and ±4.6 °C, compared to ±7.3 min and ±2.3 °C, respectively, calculated with the traditional two-step linear regression approach which is based on the mean *D_T_* values. A sensitivity analysis for the Monte Carlo trials revealed that the use of a minimum 250 × 250 data set was necessary to ensure sufficient accuracy of CI predictions. It should be noticed that, although the preceding analysis referred to first-order inactivation data and Bigelow’s model for temperature dependency of primary models kinetic parameters, the same procedure can be employed when a different primary or secondary model is used to describe kinetic data.

## Figures and Tables

**Table 1:** *D_T_* values (in min) of l-carnitine thermal inactivation.

T (°C)	From Original Data		Intentionally Expanded Error	From 500 Random Points (with the Expanded CI)	
	Mean Value	±95% CI	±95% CI	Mean Value	±95% CI
80	896.3	45.0	267.7	890.8	262.0
85	728.8	57.2	230.2	735.8	223.6
90	573.8	65.0	196.2	569.1	192.5
95	384.1	41.0	123.4	387.4	121.5
100	238.2	7.6	59.0	236.4	57.8
110	105.8	2.4	24.3	106.3	24.4
120	47.3	2.2	13.5	47.1	13.4
130	23.6	0.6	4.9	23.5	5.0

CI: Confidence Interval.

## References

[1] Taoukis P.S., Tsironi T.S., Giannakourou M.C., Tzia K., Varzakas T. (2015). Reaction Kinetics. Handbook of Food Processing and Engineering, Volume I: Food Engineering Fundamentals.

[2] Van Boekel M.A.J.S., Tijskens L.M.M., Tijskens L.M.M., Hertog M.L.A.T.M., Nicolai B.M. (2001). Kinetic modeling. Food Process Modeling.

[3] Villota R., Hawkes J.G., Heldman D.R., Lund D.B. (2007). Reaction kinetics in food systems. Handbook of Food Engineering.

[4] Van Boekel M.A.J.S. (1996). Statistical aspects of kinetic modeling for food science problems. J. Food Sci..

[5] Angelidis A.S., Papageorgiou D.K., Tyrovouzis N.A., Stoforos N.G. (2013). Kinetics of *Listeria monocytogenes* cell reduction in processed cheese during storage. Food Control.

[6] Dermesonluoglu E., Katsaros G., Tsevdou M., Giannakourou M., Taoukis P. (2015). Kinetic study of quality indices and shelf life modelling of frozen spinach under dynamic conditions of the cold chain. J. Food Eng..

[7] Huang L. (2003). Dynamic computer simulation of *Clostridium perfringens* growth in cooked ground beef. Int. J. Food Microbiol..

[8] Huang L. (2004). Numerical analysis of the growth of *Clostridium perfringens* in cooked beef under isothermal and dynamic conditions. J. Food Saf..

[9] Huang L., Vinyard B.T. (2016). Direct dynamic kinetic analysis and computer simulation of growth of *Clostridium perfringens* in cooked turkey during cooling. J. Food Sci..

[10] Sulaiman A., Soo M.J., Farid M., Silva F.V.M. (2015). Thermosonication for polyphenoloxidase inactivation in fruits: Modeling the ultrasound and thermal kinetics in pear, apple and strawberry purees at different temperatures. J. Food Eng..

[11] Sulaiman A., Soo M.J., Yoon M.M.L., Farid M., Silva F.V.M. (2015). Modeling the polyphenoloxidase inactivation kinetics in pear, apple and strawberry purees after High Pressure Processing. J. Food Eng..

[12] Giannakourou M.C., Taoukis P.S. (2003). Kinetic modelling of vitamin C loss in frozen green vegetables under variable storage conditions. Food Chem..

[13] Ávila I.M.L.B., Silva C.L.M. (1999). Modelling kinetics of thermal degradation of colour in peach puree. J. Food Eng..

[14] Bai J.W., Gao Z.J., Xiao H.W., Wang X.T., Zhang Q. (2013). Polyphenol oxidase inactivation and vitamin C degradation kinetics of Fuji apple quarters by high humidity air impingement blanching. Int. J. Food Sci. Technol..

[15] Chakraborty S., Rao P.S., Mishra H.N. (2016). Changes in quality attributes during storage of high-pressure and thermally processed pineapple puree. Food Bioprocess Technol..

[16] Colle I.J.P., Lemmens L., Tolesa G.N., Van Buggenhout S., De Vleeschouwer K., Van Loey A.M., Hendrickx M.E. (2010). Lycopene degradation and isomerization kinetics during thermal processing of an olive oil/tomato emulsion. J. Agric. Food Chem..

[17] Damasceno L.F., Fernandes F.A.N., Magalhães M.M.A., Brito E.S. (2008). Non-enzymatic browning in clarified cashew apple juice during thermal treatment: Kinetics and process control. Food Chem..

[18] Isleroglu H., Kemerli T., Sakin-Yilmazer M., Guven G., Ozdestan O., Uren A., Kaymak-Ertekin F. (2012). Effect of steam baking on acrylamide formation and browning kinetics of cookies. J. Food Sci..

[19] Jiang L., Zheng H., Lu H. (2014). Use of linear and weibull functions to model ascorbic acid degradation in Chinese winter jujube during postharvest storage in light and dark conditions. J. Food Process. Preserv..

[20] Kreyenschmidt J., Hübner A., Beierle E., Chonsch L., Scherer A., Petersen B. (2010). Determination of the shelf life of sliced cooked ham based on the growth of lactic acid bacteria in different steps of the chain. J. Appl. Microbiol..

[21] Lemmens L., De Vleeschouwer K., Moelants K.R.N., Colle I.J.P., Van Loey A.M., Hendrickx M.E. (2010). β-Carotene isomerization kinetics during thermal treatments of carrot puree. J. Agric. Food Chem..

[22] Mataragas M., Drosinos E.H., Vaidanis A., Metaxopoulos I. (2006). Development of a predictive model for spoilage of cooked cured meat products and its validation under constant and dynamic temperature storage conditions. J. Food Sci..

[23] Mohebbi M., Hasanpour N., Ansarifar E., Amiryousefi M.R. (2014). Physicochemical properties of bell pepper and kinetics of its color change influenced by *Aloe vera* and gum tragacanth coatings during storage at different temperatures. J. Food Process. Preserv..

[24] Ndoye F.T., Alvarez G. (2015). Characterization of ice recrystallization in ice cream during storage using the focused beam reflectance measurement. J. Food Eng..

[25] Nisha P., Singhal R.S., Pandit A.B. (2006). Kinetic modelling of texture development in potato cubes (*Solanum tuberosum* L.), green gram whole (*Vigna radiate* L.) and red gram splits (*Cajanus cajan* L.). J. Food Eng..

[26] Roberts J.S., Tong C.H. (2003). Drying kinetics of hygroscopic porous materials under isothermal conditions and the use of a first-order reaction kinetic model for predicting drying. Int. J. Food Prop..

[27] Schmitz-Schug I., Kulozik U., Foerst P. (2014). Reaction kinetics of lysine loss in a model dairy formulation as related to the physical state. Food Bioprocess Technol..

[28] Tsironi T., Dermesonlouoglou E., Giannakourou M., Taoukis P. (2009). Shelf life modelling of frozen shrimp at variable temperature conditions. LWT-Food Sci. Technol..

[29] Aspridou Z., Koutsoumanis K.P. (2015). Individual cell heterogeneity as variability source in population dynamics of microbial inactivation. Food Microbiol..

[30] Huang L. (2015). Dynamic determination of kinetic parameters, computer simulation, and probabilistic analysis of growth of Clostridium perfringens in cooked beef during cooling. Int. J. Food Microbiol..

[31] Koutsoumanis K., Angelidis A.S. (2007). Probabilistic modeling approach for evaluating the compliance of ready-to-eat foods with new European union safety criteria for Listeria monocytogenes. Appl. Environ. Microb..

[32] Lianou A., Koutsoumanis K.P. (2011). A stochastic approach for integrating strain variability in modeling Salmonella enterica growth as a function of pH and water activity. Int. J. Food Microbiol..

[33] Channon H.A., Hamilton A.J., D’Souza D.N., Dunshea F.R. (2016). Estimating the impact of various pathway parameters on tenderness, flavour and juiciness of pork using Monte Carlo simulation methods. Meat Sci..

[34] Evrendilek G.A., Avsar Y.K., Evrendilek F. (2016). Modelling stochastic variability and uncertainty in aroma active compounds of PEF-treated peach nectar as a function of physical and sensory properties, and treatment time. Food Chem..

[35] Giannakourou M.C., Koutsoumanis K., Dermesonlouoglou E., Taoukis P.S. (2001). Applicability of the shelf life decision system (slds) for control of nutritional quality of frozen vegetables. Acta Hortic..

[36] Giannakourou M.C., Taoukis P.S. (2003). Application of a TTI-based distribution management system for quality optimization of frozen vegetables at the consumer end. J. Food Sci..

[37] Sui X., Zhou W. (2014). Monte Carlo modelling of non-isothermal degradation of two cyanidin-based anthocyanins in aqueous system at high temperatures and its impact on antioxidant capacities. Food Chem..

[38] Wesolek N., Roudot A.C. (2016). Assessing aflatoxin B1 distribution and variability in pistachios: Validation of a Monte Carlo modeling method and comparison to the Codex method. Food Control.

[39] Efron B., Tibshirani R.J. (1993). An Introduction to the Bootstrap.

[40] Poschet F., Bernaerts K., Geeraerd A.H., Scheerlinck N., Nicolai B.M., Van Impe J.F. (2004). Sensitivity analysis of microbial growth parameter distributions with respect to data quality and quantity by using Monte Carlo analysis. Math. Comput. Simul..

[41] Poschet F., Geeraerd A.H., Van Loey A.M., Hendrickx M.E., Van Impe J.F. (2005). Assessing the optimal experiment setup for first order kinetic studies by Monte Carlo analysis. Food Control.

[42] Poschet F., Geeraerd A.H., Scheerlinck N., Nicolai M.B., Van Impe J.F. (2003). Monte Carlo analysis as a tool to incorporate variation on experimental data in predictive microbiology. Food Microbiol..

[43] Jiménez N., Bohuon P., Lima J., Dornier M., Vaillant F., Pérez A.M. (2010). Kinetics of anthocyanin degradation and browning in reconstituted blackberry juice treated at high temperatures (100–180 °C). J. Agric. Food Chem..

[44] Lima J.R., Elizondo N.J., Bohuon P. (2010). Kinetics of ascorbic acid degradation and colour change in ground cashew apples treated at high temperatures (100–180 °C). Int. J. Food Sci. Technol..

[45] Bigelow W.D., Bohart G.S., Richardson A.C., Ball C.O. (1920). Heat Penetration in Processing Canned Foods.

[46] Ball C.O. (1923). Bulletin of the National Research Council No. 37.

[47] Lammerding A.M., Fazil A. (2000). Hazard identification and exposure assessment for microbial food safety risk assessment. Int. J. Food Microbiol..

[48] Taoukis P.S., Tijkskens L.M.M., Hertog M.L.A.T.M., Nicolai B.M. (2001). Modeling the use of time-temperature indicators in distribution and stock rotation. Food Process Modeling.

[49] Prokopiou P., Goula A.M., Stoforos N.G., Taoukis P.S., Stoforos N.G., Karathanos V.T., Saravacos G.D. Thermal inactivation kinetics of l-carnitine. Proceedings of the 11th International Congress on Engineering and Food (ICEF 11).

[50] Pénicaud C., Bohuon P., Peyron S., Gontard N., Guillard V. (2012). Influence of the experimental errors and their propagation on the accuracy of identified kinetics parameters: Oxygen and temperature effects on ascorbic acid oxidation during storage. Ind. Eng. Chem. Res..

[51] Smid J.H., Verloo D., Barker G.C., Havelaar A.H. (2010). Strengths and weaknesses of Monte Carlo simulation models and Bayesian belief networks in microbial risk assessment. Int. J. Food Microbiol..

[52] Taylor J.R. (1997). An Introduction to Error Analysis.

[53] Rabinovich S.G. (2005). Measurement Errors and Uncertainties-Theory and Practice.

